# Origin of multi-level switching and telegraphic noise in organic nanocomposite memory devices

**DOI:** 10.1038/srep33967

**Published:** 2016-09-23

**Authors:** Younggul Song, Hyunhak Jeong, Seungjun Chung, Geun Ho Ahn, Tae-Young Kim, Jingon Jang, Daekyoung Yoo, Heejun Jeong, Ali Javey, Takhee Lee

**Affiliations:** 1 Department of Physics and Astronomy and Institute of Applied Physics, Seoul National University, Seoul 08826, Korea; 2 Electrical Engineering and Computer Sciences, University of California, Berkeley, California 94720, USA; 3 Department of Applied Physics, Hanyang University, Ansan 15588, Korea

## Abstract

The origin of negative differential resistance (NDR) and its derivative intermediate resistive states (IRSs) of nanocomposite memory systems have not been clearly analyzed for the past decade. To address this issue, we investigate the current fluctuations of organic nanocomposite memory devices with NDR and the IRSs under various temperature conditions. The 1/f noise scaling behaviors at various temperature conditions in the IRSs and telegraphic noise in NDR indicate the localized current pathways in the organic nanocomposite layers for each IRS. The clearly observed telegraphic noise with a long characteristic time in NDR at low temperature indicates that the localized current pathways for the IRSs are attributed to trapping/de-trapping at the deep trap levels in NDR. This study will be useful for the development and tuning of multi-bit storable organic nanocomposite memory device systems.

Recently, organic material-based nanocomposites have attracted significant attention due to their advantages in the fabrication of low-cost, low-temperature, and solution-processed electronics (such as thin-film transistors, memory devices, solar cells, and wearable sensing components) on large-area flexible platforms^1^,^2^,^3^,^4^,^5^,^6^,^7^. Among these organic electronics, high performance organic resistive memory devices with appropriate architectural designs have been widely studied in the past decade^8^,^9^,^10^. The organic nanocomposite resistive memory devices generally show unipolar-type memory characteristics, i.e., the on/off states can be set and reset at the same voltage polarity^8^. In addition, their current-voltage characteristics often show a multi-storage functionality, such as the intermediate resistive states (IRSs), which are typically attributed to a negative differential resistance (NDR) behavior^11^,^12^,^13^. Although this phenomenon has been primarily explained by the formation and rupture of conducting filaments in the active layer under a voltage bias or the charge-trapping mechanism, the strongly disordered and inhomogeneous structures have hindered the elaborated understanding of NDR and the IRSs. To exploit the multi-store functionality practically for the higher data density and the low voltage operation, thorough investigations on NDR and its derivative IRSs are necessary.

Organic nanocomposite memory devices have typically shown non-ohmic behavior at their operation voltage from 0.1 V to 3 V, which does not allow the formation of metallic conductive filaments^14^. In this regard, many research groups have concluded that various traps have a critical role in the formation of conducting paths in the organic active layers by comparing current-voltage characteristics with well-established charge transport mechanisms, such as Poole-Frenkel conduction, space charge limited current, and Fowler-Nordheim tunneling^8^,^15^,^16^,^17^. In particular, Bozano *et al*. have investigated the origin of NDR in organic nanocomposite memory devices where aluminum granules are embedded in an organic matrix (Alq_3_)^11^. As their organic memory devices showed similar electrical behaviors compared with those of inorganic metal-insulator-metal diodes where gold atoms are dispersed in a silicon oxide insulating layer by the electroforming process, Bozano *et al*. concluded that the transport and switching of organic nanocomposite memory devices are based on Coulomb blockade. According to their statements, charge carriers are trapped on metallic granules at the NDR regime forming space-charged field, so that Coulomb blockade occurs and resistance decreases (off-state). However, the off-state is usually observed in a pristine device, which is inconsistent with the previous statement^18^. Moreover, in a recently reported study for the impedance characterizations of organic nanocomposite memory devices, the Coulomb blockade-based mechanism was discluded to explain the state-independent capacitive contribution in the AC-spectra, and it was suggested that the mechanism is based on the formation of localized current pathways inside the organic material^19^. However, the causal relation between NDR and its derivative IRSs are still not clearly understood.

Under an external electric field, the time- and frequency-resolved current fluctuation information can provide key evidence for the dynamic characteristics of the resistance changes of the active memory layers. The various noise studies have provided a deep insight into the inherent charge transport mechanisms of various disordered systems, such as organic semiconductors, metal-molecule-metal junctions, inorganic filamentary memory devices, and granular systems^20^,^21^,^22^,^23^,^24^. In this regard, we have also reported the current noise and the percolative scaling behaviors in an organic nanocomposite memory structure^13^.

In this study, for further investigation, the changes of the noise characteristics in a wide range of temperatures were observed in the composite of polystyrene (PS) and phenyl-C61-butyric acid methyl (PCBM). For resistive memory systems which use nanocomposite materials, it is widely known that organic/inorganic nanoclusters embedded in organic matrices can be regarded as the charge trapping elements^11^,^16^,^17^,^18^,^25^,^26^. More specifically, the noise scaling behavior from the IRSs and the telegraphic noise in NDR were investigated at a range of temperatures from 80 K to 300 K to observe the electronic dynamics, thereby enabling a better understanding of NDR and the IRSs in organic nanocomposite memory systems. The thermally affected scaling behavior in the IRSs and telegraphic noise in the NDR regime indicated that the multi-level switching and percolation behavior in the organic nanocomposite memory devices were actually controlled by the charge trapping/de-trapping process at the deep trap levels.

## Results

### Organic nanocomposite memory and noise measurement

**Figure 1:**
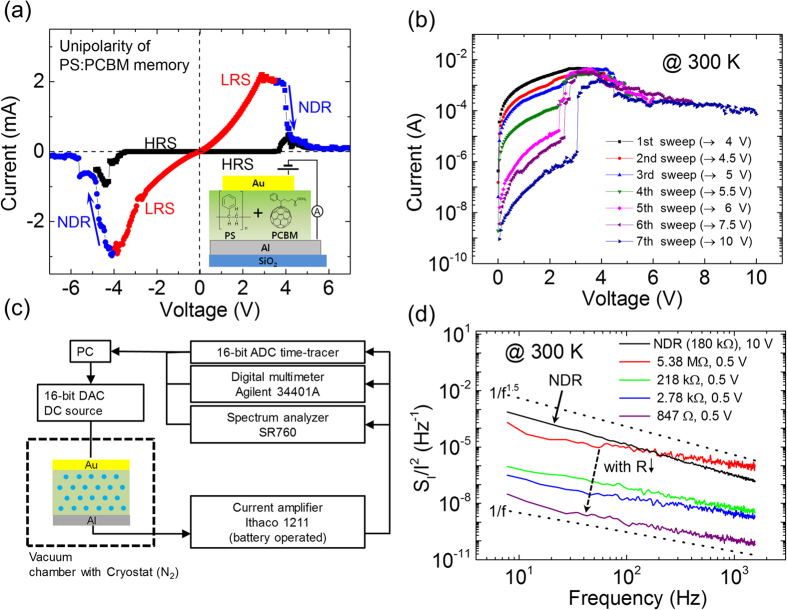
Current-voltage (I-V) and noise characteristics of PS:PCBM nanocomposite memory. **(a)** Unipolar I-V relation of a PS:PCBM nanocomposite memory device. (**b**) I-V characteristics of PS:PCBM exhibiting multi-level resistive states at room temperature. **(c)** Schematic for the noise measurement setup. **(d)** 1/f ^γ^ noise at various resistive states and NDR.

Figure 1a shows the current-voltage (I-V) relation of the PS:PCBM nanocomposite organic memory device. The schematic of the organic memory device and its I-V measurement configuration are also included in the inset. More detailed information for the PS:PCBM material preparation and device measurement are provided in the Methods section. In the I-V relation, the high resistive states (HRSs) and the low resistive states (LRSs) are presented in both voltage polarities, revealing a good on/off ratio (>10^5^) at the read voltage (|V| < 0.5 V). Usually, the HRS can be switched to the LRS by applying a voltage in the range of 3 V < |V| < 4 V. When |V| is higher than 4 V, the current starts to decrease, which corresponds to NDR. When we turned off the voltage at the stop voltage (V_stop_) in NDR, the current state returned to the HRS. Notably, the PS:PCBM organic memory showed a unipolar property, even with an asymmetrical electrode structure (Al for the bottom electrode and Au for the top electrode), which suggests that the resistive switching is mainly related to the active material region rather than the interfacial region.

Our PS:PCBM organic memory devices also showed multi-stable behavior in its I-V characteristics. Figure 1b shows I-V characteristics of the PS:PCBM resistive memory device with multi-level switching behavior. By sweeping the applied voltage from 0 V to V_stop_ and modulating V_stop_, a number of the IRSs could be approached. The resistive states ranged from 100 MΩ to 1 kΩ. Non-ohmic behavior was observed at all of the IRSs, which indicates that the conduction at low voltages (0 V < V < 3 V) was far from the conductive filamentary pathway formation (see Supplementary Fig. S1). This multi-level switching behavior could also be identified at low temperature (100 K); however, the number of accessible resistive states was less than that at room temperature (300 K) (see Supplementary Fig. S2).

Figure 1c shows a schematic describing the noise measurement setup. A digital-analog converter (DAC) was used to apply the voltage bias. A battery-powered current amplifier (Ithaco 1211) was used for converting the current noise signal into an amplified voltage signal. An analog-digital converter (ADC) and a spectrum analyzer (SR760) were used to monitor the time domain noise signal and the frequency domain noise signal (power spectral density), respectively. A digital multimeter (Agilent 34401A) was used to simultaneously measure the current and power spectral density. Because the current amplifier has a 50 kHz bandwidth with rise time of 10 μs, we measured low frequency noise in the range from 4 Hz to 1500 Hz in the frequency domain. For the low temperature experiments, a cryostat cooled with liquid nitrogen was used.

Figure 1d shows the relative power spectral densities for the current signal (*S*_*I*_*/I*^*2*^) of our memory device in the IRSs and NDR at room temperature. In the IRSs at a low bias (0.3 V), each resistive state generally showed 1/f type noise in the low frequency region (<1,500 Hz). In addition, *S*_*I*_*/I*^*2*^ decreased as the resistance decreased. On the other hand, at a high voltage (10 V) in NDR, the noise shows 1/f^1.5^ type noise (black curve). The different exponent values indicate different current conduction mechanisms present in the IRSs at a low bias and in the NDR regime at a high bias^13^,^27^,^28^. Particularly, the exponent value of 1.5 has been understood as a modulation of the local resistances of the material by a diffusing variable such as a local charge carrier density^27^,^29^,^30^.

### Percolative conducting network in PS:PCBM memory

**Figure 2:**
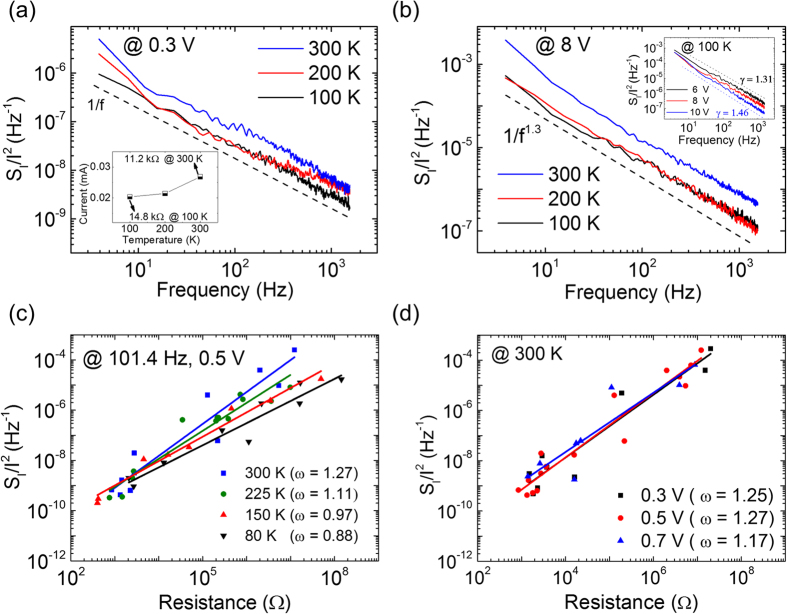
Relative power spectral density of PS:PCBM nanocomposite memory at various temperature conditions. (**a**) The relative power spectral densities at 100 K, 200 K, and 300 K in one of the IRSs. The inset indicates the corresponding current level at 100 K, 200 K, and 300 K. (**b**) The relative power spectral densities at 100 K, 200 K, and 300 K in the NDR regime (8 V bias). The inset shows the voltage dependence on the relative power spectral density at 100 K. **(c)** Power-law relationships between the relative power spectral densities and the resistance of the IRSs at 80 K, 150 K, 225 K, and 300 K with f = 101.4 Hz and 0.5 V bias. **(d)** Power-law relationship between the relative power spectral density and the resistance of the IRSs at 0.3 V, 0.5 V, and 0.7 V at 300 K with f = 101.4 Hz.

To investigate the thermal effect on electronic noise in the PS:PCBM organic memory device, *S*_*I*_*/I*^*2*^ at low temperature was studied at a low bias (0.3 V) in the IRSs and at the high biases (from 6 V to 10 V) in the NDR regime. Figure 2a and its inset show the relative noise and current level at three different temperature conditions (100, 200, and 300 K). The current level was chosen to be one of the IRSs with low resistance (11.2 kΩ at room temperature). The relative noise (*S*_*I*_*/I*^*2*^) was generally 1/f ^γ^, whereas the exponents were 0.9 < γ < 1.0. The value of γ = 1.0 ± 0.1 was consistently observed at low bias for various temperature conditions (see Supplementary Fig. S3). Moreover, the relative noise amplitude and the current level were found to decrease as temperature decreased. The magnitude of the relative noise was found to differ 2–3 times between 100 K and 300 K. In addition, the current level slightly decreased as temperature decreased (26.7 μA at 300 K decreased to 20.2 μA at 100 K, inset Fig. 2a). Figure 2b shows the temperature dependence on the relative noise in the NDR regime. The 1/f^γ^ noise with γ > 1.3 indicated that the noise in the NDR regime can be related with the discrete current levels caused by the trapping and de-trapping of charge carriers and the contribution of the diffusion process^30^,^31^,^32^. Note that the superposition of Lorentzian noise caused by traps with the characteristic time distribution can show 1/f^γ^ with γ = 1 ± 0.3^32^. In our PS:PCBM memory device at an 8 V bias, the relative noise maintained 1/f noise with γ ~ 1.3. In addition, approximately 3 times lower relative noise was observed at 100 K and 200 K than at 300 K. By varying the voltage in the NDR regime, we also observed that γ increased with increasing voltage from 1.31 (at 6 V) to 1.46 (at 10 V) (inset of Fig. 2b). The increase of γ can be explained by the domination of trapping/de-trapping processes in deep trap levels with a higher voltage bias^13^.

As mentioned above, by setting a higher V_stop_, the IRSs with higher resistance can be approached, as the current pathways with a reduced conductivity are fixed (various IRSs shown in Fig. 1b). Thus, the voltage bias modulation in NDR can lead to the various resistive states by current pathway deformation and fluctuation. These current pathways could be identified as conductive filaments, such as metal ion inclusions or phase transition of the active layer^14^,^23^. However, the nonlinear (non-ohmic) and the unipolar I-V relation of the PS:PCBM memory device was not consistent with those arguments; rather, the I-V characteristic indicates that the current pathways are related to the conduction along trap-free (or trap-filled) regions. These various current pathways for each IRS can be regarded as a current distribution through the resistance network of a percolation system. Because traps play the part of current bridges, the trap sites can be considered to be elementary bonds or sites in a percolation theory. In the conductive percolating network, the relative power spectral density and resistance exhibits a scaling behavior in the condition where the conductive phase fraction *φ* is higher than a critical conductive phase fraction *φ*_*c*_, described as^33^


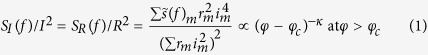



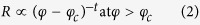


where *r*_*m*_ denotes the m-th primitive resistor in the percolation network, *i*_*m*_ is the fractional current flowing through *r*_*m*_, 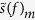 is the spectral density of fractional noise of *r*_*m*_, and *R* is the total resistance of the percolating network. Note that in the above relations, the resistance *R* and relative power spectral density *S*_*I*_*/I*^*2*^ diverge as *φ* approaches *φ*_*c*_ from the conductive side. If the percolating network has smaller current pathways with lower *φ* (when approaching *φ*_*c*_), a large amount of current becomes concentrated on a few current pathways. Therefore, the higher relative noise from the network can be induced. Because it is difficult to obtain the exact value of *φ* from experiments, the scaling behaviors of *S*_*I*_*/I*^*2*^ and *R* can be combined to the experimentally obtainable scaling relation as


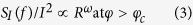


where the exponent *ω* denotes *κ/t*. To investigate the relationship between *S*_*I*_*/I*^*2*^ and *R*, each IRS was approached by modulating V_stop_ at the NDR regime, and then the power spectral density and average current were measured simultaneously. We examined the relationship between the *S*_*I*_*/I*^*2*^ and *R* by varying both the temperatures and the voltage biases. Figure 2c shows that PS:PCBM organic memory devices exhibit a scaling behavior between the relative power spectral density and resistance. As the temperature decreases from 300 K to 80 K, ω was observed to decrease from 1.27 to 0.88. A similar ω value (1.22) at 300 K was also observed from other devices (see Supplementary Fig. S4). The ω value of the PS:PCBM memory device for 300 K can be comparable to ω = 0.95 measured for the polyimide (PI):PCBM organic nanocomposite memory device at room temperature in the previous study^13^. Although PCBM concentration, monomer type, and degree of polymerization are different between PS and PI, both PS:PCBM and PI:PCBM showed similar resistive switching and noise characteristics. The minor difference of ω between PS:PCBM and PI:PCBM is attributed to the geometrical differences of the trap distribution and the energy disorder between the polymers and PCBM. Note that ω = 1.8 was reported for a conductive filament type nickel oxide resistive memory device at room temperature^23^. The relative noise in the LRS, which has abundant current pathways, was not significantly affected by the temperature conditions. In contrast, the relative noise in the HRS, which lacks current pathways, was greatly changed by the temperature conditions, showing over a 10^2^ magnitude difference between 300 K and 80 K.

The change of ω at different temperature conditions can be attributed to the geometrical variation of the current pathways. From a relation between electrical current and mobility (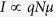), fluctuations in the electrical current can be written as 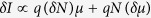, where *q* is the charge of a charge carrier, *N* is the number of charge carriers, and *μ* is the mobility. Accordingly, mobility fluctuation (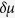) and number fluctuation (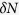) have been considered as the sources of 1/f noise^31^,^32^,^34^. Mobility fluctuation is primarily caused by charge scattering, and number fluctuation is determined by capture or emission of electrons at trap/scattering centers. Although the major source for the 1/f noise in PS:PCBM is uncertain here, we can explain the lower *ω* at low temperature with both mobility and number fluctuations. If the number of current pathways is lower at the higher resistive state, then the pathways for charge transport are more limited and become thinner. Charge carriers with higher thermal energy would undergo surface scattering (mobility fluctuation) in the thin current pathway because charge carriers in organic polymers have longer inelastic mean free path at higher temperature^35^,^36^. If the width of the current pathway gets thinner, surface scattering would occur more frequently. On the other hand, charge carriers with higher thermal energy would be more likely to be captured/emitted at shallow trap centers (number fluctuation). In any case, the geometrical formation of current pathways is important in the different scaling behaviors at the different temperatures. Meanwhile, ω was unchanged under varying low bias voltages at room temperature (Fig. 2d), indicating that the resistance fluctuation in the PS:PCBM percolating network remains the same under different voltage biases, i.e., the scaling behavior is induced by purely geometrical formation of current pathways in the low bias range^23^.

### Telegraphic noise in the NDR regime and current pathway fluctuation

**Figure 3:**
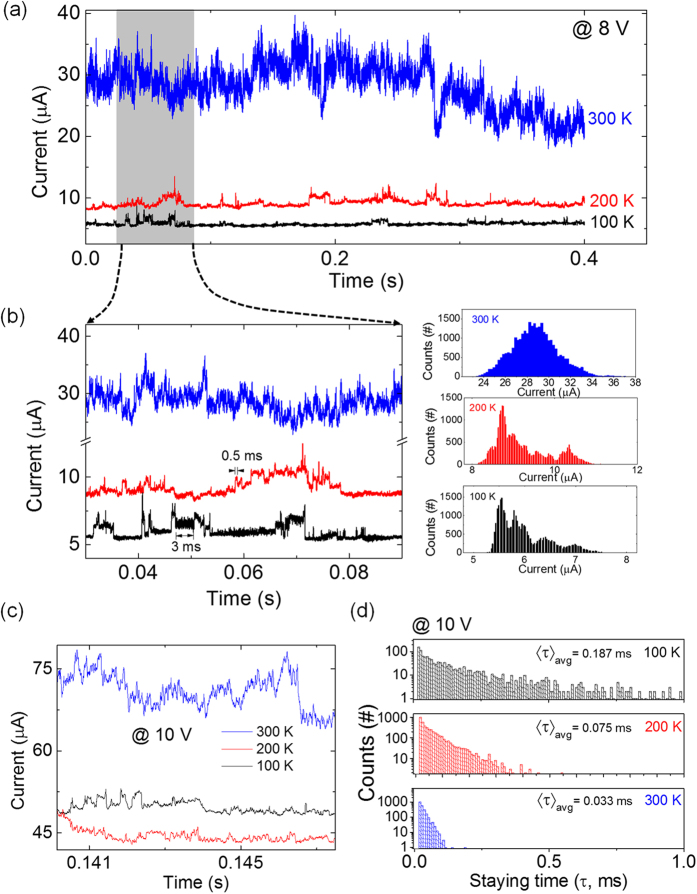
Telegraphic noise of PS:PCBM nanocomposite memory at NDR. Time traces of currents at 100 K, 200 K, and 300 K in the NDR regime (8 V bias) for **(a)** 0.4 s and **(b)** 50 ms. The right inset in **(b)** is the corresponding current histograms at 100 K, 200 K, and 300 K time traces for 50 ms. **(c)** Time traces of the currents at 100 K, 200 K, and 300 K at 10 V bias and **(d)** distribution of the dwell times for telegraphic noise at 100 K, 200 K, and 300 K with 10 V bias.

We studied the time domain signal of noise in NDR with varying temperatures from 100 K to 300 K. In the low bias regime where 1/f noise is found (<1 V), the time trace data showed a single peak distribution of current values (see Supplementary Fig. S5). In the NDR regime (>4 V), however, the time trace signal usually exhibited alterations of the current levels, which can be called telegraphic noise (Fig. 3). At the bias of 8 V in the NDR regime, the time trace data were measured at 100 K, 200 K, and 300 K. At 300 K, the current noise showed a large fluctuation with a magnitude of approximately 10 μA. In contrast, the fluctuating noise was drastically reduced and showed longer dwell times at 200 K and 100 K. In addition, the noise at 300 K showed alternating current plateaus with a much shorter dwell time of approximately tens of microseconds (see Supplementary Fig. S6). The histogram graphs at the right of Fig. 3b indicate the distribution of current values within 60 ms at the temperatures of 100 K, 200 K, and 300 K. At 300 K, because the characteristic time for the current plateaus transition was too small, the distribution of current values appeared to have one peak. As the temperature decreased to 200 K and 100 K, several distinct peaks could be observed in the current distribution. Compared with the time traces for 200 K and 100 K, the data for 100 K showed a much longer dwell time of the current level transition. The telegraphic noise at NDR was also affected by the applied voltage, as the increasing voltage led to more frequent current level transitions at 100 K (see Supplementary Fig. S7).

Usually, two-level random telegraph noise is characterized by estimating the characteristic times of capture/emission^37^,^38^. However, because the telegraphic noise of the PS:PCBM memory device showed an alternation in the multiple current states, we estimated the representative dwell time of this telegraphic noise as the average of all the dwell times (τ) for which current states maintain. We measured the telegraphic noise with a bias of 10 V at various temperature conditions (Fig. 3c). Because the telegraphic noise at 10 V showed a shorter dwell time than that at 8 V, an adequate amount of τ sampling could be achieved. Then, to evaluate the change of the average of the dwell times (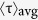) in the temperature conditions, we measured τ and plotted the distribution on a semi-log scale (Fig. 3d). In Fig. 3d, the flections at the border of different slopes of the distributions were observed at 100 K and 200 K. These flections can be attributed to the contributions of different characteristic times of traps which are major noise sources in the time traces. 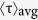 decreased as the temperature increased, with values of 0.187 ms, 0.075 ms, and 0.033 ms for 100 K, 200 K, and 300 K, respectively. Here, the longer dwell times at lower temperature and the lower voltage bias indicate that the noise was induced by a voltage driven and thermally activated trapping/de-trapping process at the deep trap levels^39^.

**Figure 4:**
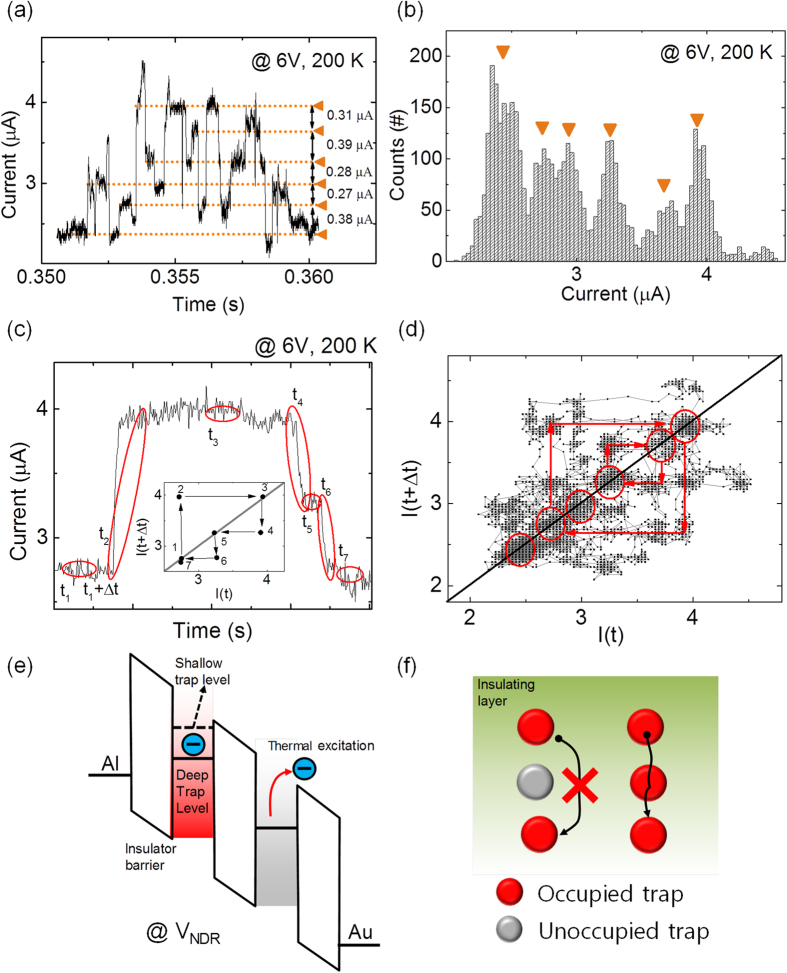
Reversible access of the resistive states and schematics of the current pathway formation and removal process. **(a)** Well-separated current plateaus at 6 V bias and 200 K and **(b)** the corresponding current histogram. **(c)** Explanation of the Time Lag Plot (TLP). **(d)** TLP of **(a)**. **(e)** Schematic of the current pathway removal process with NDR modulation. The blue colored circles indicate the electrons that are localized in the deep trap levels or escaping the deep trap levels. **(f)** Schematic of current pathway formation and removal by the charge trapping/de-trapping at the deep trap levels.

Figure 4a,b show six of the well-separated current plateaus at the bias of 6 V at 200 K. These current plateaus have a nearly regular current interval of approximately 0.3 μA. Each current plateau was reversibly accessible during the discrete current alteration process. This reversible access of current states indicates that the telegraphic noise occurs in the stationary structure. For a number of traps *n*, the number of current states can be *2*^*n *^38; thus, at least 3 traps contributed to the random telegraphic noise, as shown in Fig. 4a. To better visualize the dynamics of the transition between the current states, the behavior is shown in a time lag plot (TLP)^40^,^41^. Note that a TLP is a simple but effective analysis method for understanding both the complex waveforms and the statistical behaviors of telegraphic noise. A TLP can be drawn by plotting points on a 2-dimensional graph, where the x- and y-axis are set to the current value at time *t* and *t* +* Δt*, respectively; one can track the transitions between the current states from this dragging feature (Fig. 4c). For our study, we set *Δt* = 50 μs because the transition time was estimated to be 10–20 μs. Figure 4d shows the TLP for the data of Fig. 4a. For the diagonal element, six current states were observed, as shown in the raw time trace (Fig. 4a). The non-diagonal points indicate the transition between current states, i.e., the upper points indicate the transition from the lower current state to the higher current state and vice versa.

The telegraphic noise shown in the PS:PCBM organic nanocomposite memory device can be understood as the current paths fluctuation induced by charge trapping and de-trapping in the deep trap levels distributed in the organic polymer. A schematic illustrating the current pathway formation is presented in Fig. 4e. The white boxes indicate the gap between the highest occupied molecular orbital (HOMO) and the lowest unoccupied molecular orbital (LUMO) of the insulating matrix (PS). As the trap centers are distributed in the insulating matrix, charge carriers can be trapped and localized in the deep trap energy levels due to a high trap energy barrier relative to the conduction level of the insulating matrix. If the deep trap levels are already occupied by charge carriers, charge carriers can easily pass through the shallow trap levels above the deep trap levels *via* trap-assisted tunneling or the Poole-Frenkel conduction process (Fig. 4f)^13^,^42^. As a result, the neighboring traps with the occupied deep trap levels can form a current pathway. At the high voltage bias, charge carriers trapped in the deep trap levels can be unstable and detrapped due to the lowered trap barrier and thermal excitation. When the deep trap level is unoccupied, the current pathway between traps is cut off. If the unoccupied deep trap levels are reoccupied, the current pathways can be reformed. Thus, the telegraphic noise in the NDR regime can be understood as the fluctuation of the current pathways due to the trapping/de-trapping process at the deep trap levels. Due to the energy distribution of the deep trap levels in the PS:PCBM layer^13^, more charge carriers in the deeper trap levels would be unstable as the higher bias is applied, resulting in a decrease in the number of current pathways. Furthermore, the exponent value of 1.5 at the high voltage bias in Fig. 1d can be explained by the diffusion process caused by a gradient of the local number density of charge carriers trapped in the deep trap levels.

In summary, we present the noise characteristics of the organic nanocomposite (PS:PCBM) memory under various temperature conditions. The geometrical formation of constricted current paths can exhibit percolation behavior in the form of 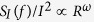. The suppression and clear appearance of the telegraphic noise in NDR at low temperature indicates that the charge trapping/de-trapping process at the traps is the principal cause of NDR, and this process results in the intermediate resistive states at low voltage bias via the current pathway formation. The trap formation and distribution in the nanocomposite resistive memory device system will be important in next-generation resistive memory devices; this study will provide a better understanding of resistive memory devices and promote the development of more practical and sophisticated resistive memory devices.

## Methods

### Device Fabrication

For the nanocomposite organic memory material (PS:PCBM), 16 mg of polystyrene (PS) and 2.5 mg of phenyl-C61-butyric acid methyl ester (PCBM) were dissolved in 2 mL of chlorobenzene. The thermally grown SiO_2_ (270 nm thick)/Si substrate was sequentially cleaned with acetone, 2-propanol, and deionized water in sonication for 10 min. Al bottom electrode lines with 100 μm width and 30 nm thickness were deposited using shadow mask patterning. The bottom Al electrodes were exposed to UV-ozone for 10 min to enhance the film uniformity^7^. The prepared nanocomposite memory layer was spin-coated onto the substrate and then annealed on a hot-plate in N_2_ at 60 °C for 10 min, followed by exposure of the bottom electrodes using an acetone-soaked swab and then heating at 120 °C for 60 min in N_2_. Next, Au top electrodes with 100 μm width and 30 nm thickness were deposited onto the active memory layer. The spin-coated PS:PCBM film was confirmed to be well-blended via photoluminescence measurements (see Supplementary Fig. S8).

### Device measurement

The I-V characteristics of the devices were measured using a semiconductor parameter analyzer (Keithley 4200 SCS) and a probe station system (JANIS Model ST-500). For the noise measurement, a spectrum analyzer (Stanford Research SR780) and a ground-isolated 16-bit analog-digital converter (ADC) were used to measure the current noise characteristics in the frequency and time domains, respectively. A battery-powered low-noise current amplifier (Ithaco 1211) was used for converting and amplifying the current noise signal into a voltage signal. A 16-bit digital-analog converter (DAC) was used to apply a voltage bias. A digital multimeter (Agilent 34401A) was used to obtain the average electric current coinciding with the noise power spectral density measurement. All the measurements were performed in a vacuum environment.

## Additional Information

**How to cite this article**: Song, Y. *et al*. Origin of multi-level switching and telegraphic noise in organic nanocomposite memory devices. *Sci. Rep*. **6**, 33967; doi: 10.1038/srep33967 (2016).

## Supplementary Material

Supplementary Information
