# Supplementary material for: Origin of multi-level switching and telegraphic noise in organic nanocomposite memory devices
Source: Sci Rep. 2016 Sep 23;6:33967. doi: 10.1038/srep33967 (PMC5034279; doi:10.1038/srep33967)
Supplement: Supplementary Information [file srep33967-s1.doc]

Supplementary Information

Origin of multi-level switching and telegraphic noise

in organic nanocomposite memory devices

Younggul Song^1^, Hyunhak Jeong^1^, Seungjun Chung^1,2^, Geun Ho Ahn^2^, Tae-Young Kim^1^, Jingon Jang^1^, Daekyoung Yoo^1^, Heejun Jeong^3^, Ali Javey^2^ & Takhee Lee^1^*

*^1^Department of Physics and Astronomy, and Institute of Applied Physics, Seoul National University, Seoul 08826, Korea.*

*^2^Electrical Engineering and Computer Sciences, University of California, Berkeley, California 94720, USA.*

*^3^Department of Applied Physics, Hanyang University, Ansan 15588, Korea.*

*Correspondence and requests for materials should be addressed to T. L. [(tlee@snu.ac.kr)](mailto:(tlee@snu.ac.kr)).

**1. Non-ohmic I-V characteristics of intermediate resistive states**

The I-V characteristics of the intermediate resistive states (IRSs) of PS:PCBM nanocomposite memory devices are shown in Fig. S1. In linear scale graph (Fig. S1(a)), all the IRSs showed non-ohmic behavior in 0.2−3 V, i. e., the main conduction mechanism of PS:PCBM memory would not be a conductive filamentary conduction regardless of the resistive states^S1,S2^. In the log-log scale plot (Fig. S1(b)), the I-V relation around 0.1 V was ohmic (I  V). Increasing the voltage bias, I-V relations become non-ohmic (I  V^β^, β > 1). At the voltage close to the switching voltage, β values were in the range between 1.3 < β < 3. This kind of I-V relation can be related to the space charge limited current conduction or Poole-Frenkel conduction mechanism, which are related to the trap-mediated conduction^S3,S4^.

**Figure S1.** (a) linear-scale I-V and (b) log-log scale I-V characteristics of IRSs.

**2. Low temperature multi-stable I-V characteristics**

The PS:PCBM organic nanocomposite resistive memory also shows the multi-stable resistive switching characteristics at low temperature (Fig. S2). Unlike the I-V characteristics at room temperature, however, the multi-stable behavior wasn’t much various at low temperature. This phenomenon seems to be attributed to the limitation of thermal excitation of trapped charges in deep traps at low temperature.

**Figure S2.** Multi-stable I-V characteristics of PS:PCBM memory devices at a low temperature (80 K).

**3. 1/f noises of the IRSs at various temperatures**

In Figure S3, relative current power spectral densities at IRSs were measured at low bias (0.5 V) and the different temperatures (80 K, 150 K, 225 K, and 300 K). The noise at low bias showed 1/f^γ^ noise form with 0.9 < γ < 1.1 at low frequency (0 Hz < *f* < 1500 Hz), regardless of temperature.

**Figure S3.** 1/f noises were observed at various IRSs at low bias (0.5 V) at (a) 80 K, (b) 150K, (c) 225 K, and (d) 300 K.

**4. Power-law behavior between S_I_/I^2^ and *R***

Figure S4 shows a power-law behavior between *S_I_/I^2^* and *R* observed from other PS:PCBM memory device. The scaling exponent ω was found to be 1.22.

**Figure S4.** Power-law relationship between *S_I_/I* and *R* observed from other PS:PCBM memory device at *f* = 101.4 Hz and 0.5 V bias conditions.

**5. Time trace and 1/f noise at the low bias**

At the low bias regime, a time trace shows a single peak distribution of current values (Fig. S5(a) and inset). In the same resistive state, the relative power spectral density shows 1/f noise with γ = 0.98.


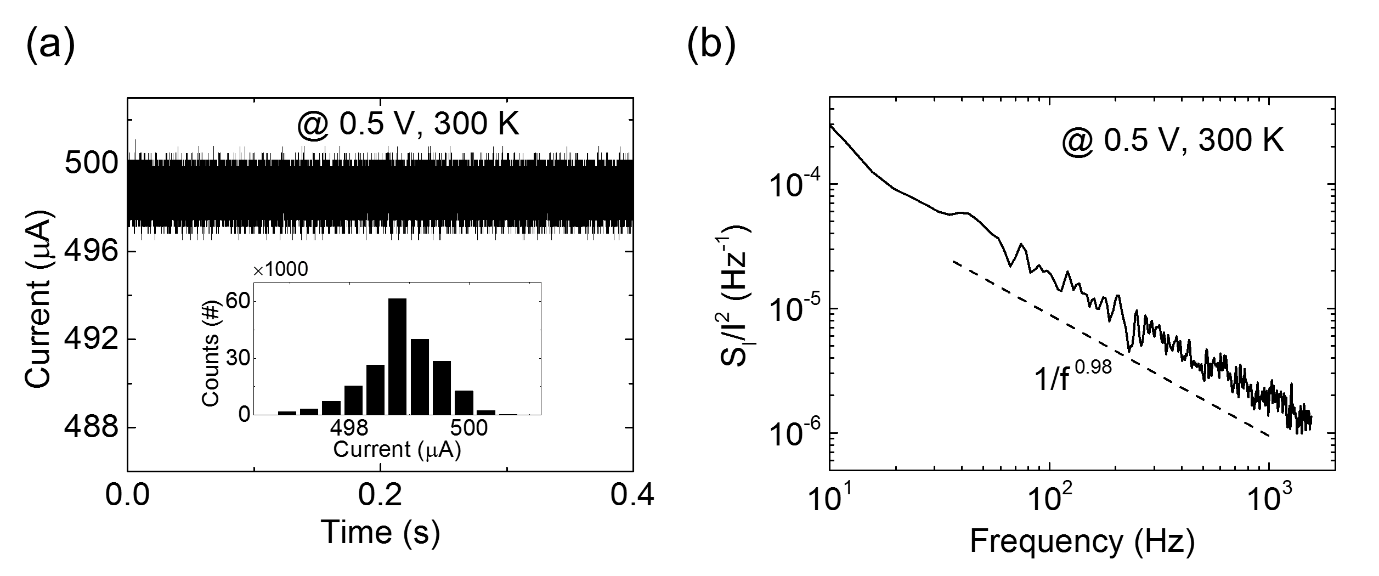


**Figure S5.** (a) Time trace and (b) relative power spectral density at 0.5 V bias and 300 K. The inset in (a) shows a current distribution of (a).

**6. Short time scale of trapping/de-trapping events at room temperature**

**Figure S6.** Time trace of current at 300K in the NDR regime (8 V) for 2 ms.

**7. Current fluctuation changes at high bias in low temperature (100 K)**

If the switching mechanism of PS:PCBM resistive memory originates from trap sites, the telegraphic noise would be affected by not only temperature, but also by an applied voltage bias, because the applied voltage bias will give an influence on the trapping/de-trapping process by the lowered trap barrier. At 100 K, the large telegraphic fluctuation was observed in PS:PCBM memory devices as increasing the voltage from 6 V to 10 V (Fig. S7). Thermal excitation of charges trapped in the deep trap levels was not enough at 6 V for the overcoming the trap barrier. As increasing the voltage to 10 V, otherwise, thermal excitation was high enough to overcome the trap barrier, resulting in the telegraphic noise.

**
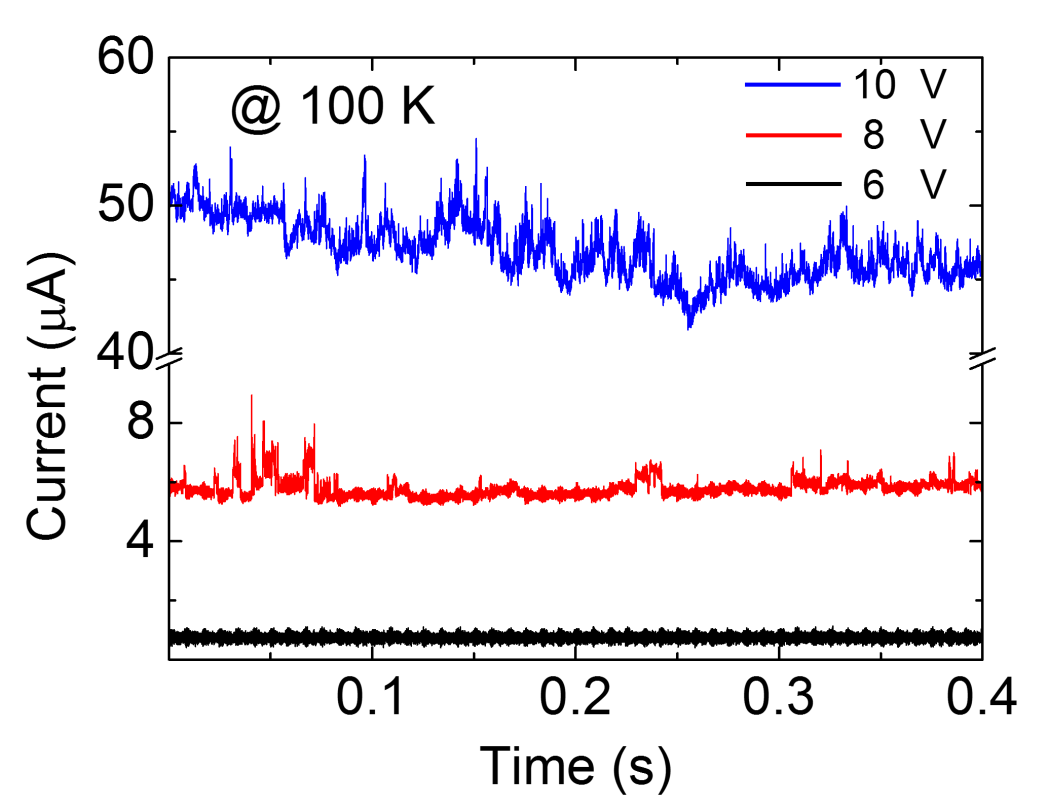
**

**Figure S7.** Time traces of currents at 6 V, 8 V, and 10 V in 100 K for 0.4 s.

**8. Photoluminescence measurement of PS:PCBM**

In Figure S8, photoluminescence (PL) spectra were measured on neat PS and PS:PCBM films (0 wt% and 13.5 wt% of PCBM in PS). A 514 nm laser was used as a incidence beam. For only PS, no PL peak was observed in the range between 1.31 eV and 2.28 eV. When PCBM was added to PS, three of PL peaks were observed. As the first sholder peak (1.558 eV) and the middle peak (1.702 eV) were typically observed from neat PCBM film^S5^, PS:PCBM film was observed to be well blended nanocomposition. Third peak (1.97 eV) at higher PCBM concentration can be related to the photo-induced interaction between PS and PCBM, such as charge transfer^S6^.

**Figure S8.** Photoluminescence peak of PS film and PS:PCBM film.

**Reference**s

1. Kwon, D.-H. *et al.* Atomic structure of conducting nanofilaments in TiO_2_ resistive switching memory. *Nat. Nanotechnol.* **5,** 148–153 (2010).
2. Brütting, W., Berleb, S. & Mückl, A. G. Space-charge limited conduction with a field and temperature dependent mobility in Alq light-emitting devices. *Synth. Met.* **122,** 99–104 (2001).
3. Chen, J.-C., Liu, C.-L., Sun, Y.-S., Tung, S.-H. & Chen, W.-C. Tunable electrical memory characteristics by the morphology of self-assembled block copolymers: PCBM nanocomposite films. *Soft Matter* **8,** 526–535 (2012).
4. Lang, D. V., Chi, X., Siegrist, T., Sergent, A. M. & Ramirez, A. P. Bias-dependent generation and quenching of defects in pentacene. *Phys. Rev. Lett.* **93,** 076601 (2004).
5. Loi, M. A. *et al.* Charge transfer excitons in bulk heterojunctions of a polyfluorene copolymer and a fullerene derivative. *Adv. Funct. Mater.* **17,** 2111–2116 (2007).
6. He, G., Li, Y., Liu, J. & Yang, Y. Enhanced electroluminescence using polystyrene as a matrix. *Appl. Phys. Lett.* **80,** 4247–4249 (2002).
